# Alternating irinotecan with oxaliplatin combined with UFT plus leucovorin (SCOUT) in metastatic colorectal cancer

**DOI:** 10.1038/sj.bjc.6604499

**Published:** 2008-08-05

**Authors:** H Y Sheikh, J W Valle, T Waddell, K Palmer, G Wilson, A Sjursen, O Craven, R Swindell, M P Saunders

**Affiliations:** 1 Department of Clinical Oncology, Christie Hospital, Manchester, UK; 2 Department of Medical Oncology, Christie Hospital, Manchester, UK; 3 Department of Medical Statistics, Christie Hospital, Manchester, UK

**Keywords:** irinotecan, metastatic colorectal cancer, oxaliplatin, tegafur–uracil, UFT

## Abstract

Tegafur–uracil (UFT) plus leucovorin® (LV, folinic acid) with alternating irinotecan and oxaliplatin were effective and well tolerated in patients with metastatic colorectal cancer (mCRC) in a phase I study. This study expanded the maximum tolerated dose group. Patients aged ⩾18 years had histologically confirmed, inoperable, previously untreated, measurable mCRC. Patients received irinotecan 180 mg m^−2^ on day 1, oxaliplatin 100 mg m^−2^ on day 15 and UFT 250 mg m^−2^ plus LV 90 mg on days 1–21 every 28 days. The phase I/II study comprised 45 patients, 29 at the maximum tolerated dose (MTD). The response rate in 38 evaluable patients was 63% (95% confidence interval (CI): 49–80). Median time to progression and overall survival were 8.7 months (95% CI: 7.9–10.4) and 16.8 months (95% CI: 9.6–25.3), respectively. In the MTD group, one patient had grade 3 leucopaenia; one had grade 3 neutropaenia; three had grade 3 diarrhoea; and one had grade 3 neurotoxicity. No hand–foot syndrome grade >1 was seen. In total, 67% of eligible patients received second-line therapy. UFT plus LV with alternating irinotecan and oxaliplatin is an efficacious first-line treatment for mCRC, with minimal neurotoxicity and hand–foot syndrome.

Patients with unresectable metastatic colorectal cancer (mCRC) have a poor prognosis in the absence of effective chemotherapy. The foundation of treatment for these patients is 5-fluorouracil (5-FU) with leucovorin® (LV), either alone or in combination with oxaliplatin (FOLFOX) or irinotecan (FOLFIRI). Many patients with mCRC receive only first-line treatment (Table 1), with some refusing second-line therapy and preferring to preserve their quality of life, whereas others are not offered this option as they are considered unfit as a result of disease progression.

Optimising first-line treatment is essential in mCRC. As exposure to three active agents, rather than second-line therapy itself, appears to predict improved survival (Grothey and Sargent, 2005), ‘up-front’ administration of three effective drugs may be the most effective way to improve outcomes. Consequently, several groups have investigated the FOLFOXIRI combination (5-FU, oxaliplatin and irinotecan) in patients with mCRC (Masi *et al*, 2004; Souglakos *et al*, 2006; Falcone *et al*, 2007). Results from phase III studies have been conflicting, however, with Falcone *et al* (2007) demonstrating better outcomes for patients treated with FOLFOXIRI compared with FOLFIRI, and Souglakos *et al* (2006) reporting no significant difference between the two regimens. Nonetheless, extremely promising overall survival (OS) rates were reported in these studies (22.6 months and 21.5 months, respectively), and a triple-drug approach holds great promise, provided the triplet regimen is tolerable.

Toxicity is a significant problem with 5-FU-based regimens. Intravenous (i.v.) bolus 5-FU is associated with considerable myelosuppression (Meta-Analysis Group In Cancer, 1998). Continuous infusion 5-FU, although less toxic, requires venous access using a tunnelled central line and portable infusion pumps, which are inconvenient for patients, while indwelling catheters can cause infection and thrombosis (Puig-la Calle *et al*, 1996; Verso and Agnelli, 2003). The oral fluoropyrimidine tegafur–uracil (UFT) is a convenient and well tolerated alternative to i.v. 5-FU. In phase III studies, UFT with LV had equivalent efficacy compared to that of i.v. 5-FU/LV, but with significantly better tolerability (Carmichael *et al*, 2002; Douillard *et al*, 2002). In both studies, UFT with LV was associated with significantly fewer haematological adverse events, including febrile neutropaenia, than bolus 5-FU/LV. In addition, patients in the UFT with LV group had a significantly lower incidence of stomatitis and other gastrointestinal events than 5-FU/LV. A number of small studies have shown that UFT can be combined with oxaliplatin (Feliu *et al*, 2004; Rosati *et al*, 2005; Bennouna *et al*, 2006; Bajetta *et al*, 2007a) or irinotecan (Mendez *et al*, 2005; Delord *et al*, 2007; Bajetta *et al*, 2007a), both combinations being effective and well tolerated in first-line mCRC.

The present phase I/II study, SCOUT (study of CPT-11, oxaliplatin, UFT triple therapy), evaluated the efficacy and tolerability of UFT with LV plus alternating irinotecan and oxaliplatin in chemonaive patients with mCRC. We hypothesised that alternating oxaliplatin and irinotecan would allow patients to benefit from concurrent treatment with all three drugs as soon as they were diagnosed with metastatic disease, while allowing them to recover from adverse events associated with each drug before it was administered again. Results from the phase I study have been published in full (Sheikh *et al*, 2007). Here we present results for the expanded group of patients treated at the maximum tolerated dose (MTD) and overall results from the phase I/II study.

## Materials and methods

### Study design and patients

This single-centre, open-label, non-randomised phase I/II study was conducted at the Christie Hospital (Manchester, UK) on patients with histologically confirmed, metastatic adenocarcinoma of the colon or rectum, with inoperable, measurable metastatic disease. Inclusion and exclusion criteria have been described previously (Sheikh *et al*, 2007). In brief, patients were aged ⩾18 years, had no prior chemotherapy for metastatic disease and a World Health Organization performance status of 0–2. All patients had adequate organ and haematological function.

The trial was conducted with full approval of the local ethical committee, according to accepted standards of good clinical practice, and in agreement with the latest version of the Declaration of Helsinki. All patients provided written informed consent.

### Treatment

In the phase I study, patients received irinotecan 180 mg m^−2^ as a 90 min infusion on day 1 of the 28-day cycle, oxaliplatin 85–100 mg m^−2^ as a 2 h infusion on day 15 and UFT capsules 200–300 mg m^−2^ d^−1^ taken orally with LV 90 mg d^−1^ in three divided doses on days 1–21. The MTD was irinotecan 180 mg m^−2^, oxaliplatin 100 mg m^−2^ and UFT 250 mg m^−2^ plus LV 90 mg. Prophylactic antiemetics (dexamethasone 8 mg and ondansetron 8 mg) were administered intravenously with the irinotecan and oxaliplatin infusions and thereafter orally for 48 h.

Chemotherapy was administered for at least 8 weeks (two cycles) before radiological reassessment, unless a criterion for study discontinuation was met. Patients remained on treatment until clinical or radiological progression, the occurrence of unacceptable or cumulative toxicity or withdrawal was requested by the patient or investigator. Treatment was continued for a further 2 months in patients with signs of clinical benefit (stable disease or complete or partial response) up to 6 months. Selected patients could continue treatment for longer than 6 months at the investigator's discretion, and could receive further treatment with the SCOUT regimen or another treatment of the investigator's choice. Re-treatment with SCOUT was only allowed in patients who had not progressed during the initial treatment period.

### Dose reductions and delays

Chemotherapy was delayed by 1 week in patients with neutropaenia grade ⩾2 or platelets <100 × 10^9^ l^−1^ (<75 × 10^9^ l^−1^ for oxaliplatin). If there was more than one delay or if a delay lasted ⩾2 weeks, doses of irinotecan and oxaliplatin were reduced by 20% and the daily UFT dose was reduced by one capsule (100 mg). Treatment continued at the lower dose for subsequent cycles unless further toxicity occurred. If a further delay for myelotoxicity occurred, 50% reduction was made to the original irinotecan and oxaliplatin doses. Withdrawal of the patient from the study was considered if their performance status had deteriorated.

Delayed diarrhoea was treated early and aggressively with loperamide, with the addition of oral ciprofloxacin if it persisted for >24 h. However, after grade 3/4 diarrhoea, treatment was delayed until complete recovery, and then resumed at 80% of the irinotecan dose and with the UFT daily dose reduced by one capsule. If diarrhoea of any grade had not resolved by the next cycle, treatment was delayed by 1 week. If further grade ⩾3 diarrhoea occurred, irinotecan was reduced to 50% of the original dose and the daily UFT dose was reduced by two capsules. Oxaliplatin was omitted for grade ⩾3 paraesthesia of hands or feet and dysaesthesia in the throat. Any significant deterioration in liver or renal function was investigated by ultrasound examination to rule out reversible biliary or renal outflow obstruction.

### Response and toxicity evaluation

Patients were assessed clinically every 14 days and a full blood count and biochemistry profile performed. Serum tumour carcinoembryonic antigen (CEA) measurements were performed monthly if raised at baseline. Toxicities were recorded at 2-weekly visits according to the National Cancer Institute Common Toxicity Criteria (version 2) and the dose-limiting toxicities at 1 month, that is, diarrhoea, lethargy and vomiting, used to decide the dose escalation schedule during the phase I study, as reported previously (Sheikh *et al*, 2007).

Radiological assessment by CT scan was performed after two cycles using the Response Evaluation Criteria in Solid Tumors (RECIST) (Therasse *et al*, 2000). All scans were performed by a dedicated gastrointestinal radiologist who provided measurements of marker lesions. From these measurements, response was confirmed by two research fellows. In cases of disagreement, scans were reviewed by the principal investigator. Only patients receiving two full 28-day cycles were assessable for the primary endpoint, that is, the objective response rate (ORR), defined as the number of patients achieving a partial or complete response at 8 weeks.

## Results

### Patient characteristics

Overall, 45 patients were treated, 29 of whom received the MTD. Patient demographics and clinical characteristics are shown in Table 2. In total, seven patients did not receive two cycles of SCOUT and were not evaluable for response assessment; four of these patients were in the MTD group. The evaluable population therefore comprised 38 patients in total and 25 patients treated at the MTD. Overall, five patients were not assessable for time to progression (TTP), two of whom were treated at the MTD (three patients died before completing an assessment scan after two cycles and two withdrew for psycho-social reasons). All patients were assessable for toxicity and OS. A majority of patients had multiple lesions; only six patients had disease confined to the liver.

### Response to treatment

Response to treatment is shown in Table 3.

**Figure 1:**
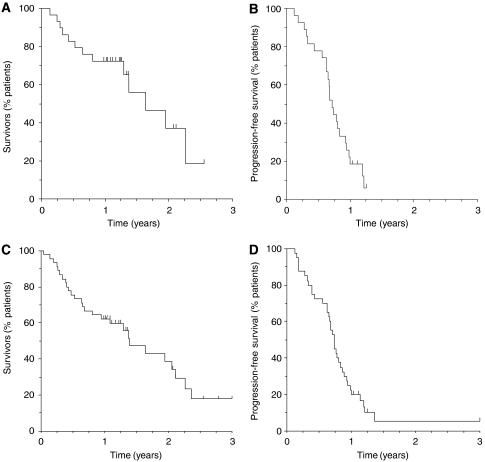
Kaplan–Meier curves for (**A**) overall survival (*n*=29) and (**B**) time to progression (*n*=27) for patients treated at the MTD, and (**C**) overall survival (*n*=45) and (**D**) time to progression (*n*=40) for all patients in the phase I/II study.

An ORR of 68% (95% CI: 46–85%) and a disease-control rate of 100% (95% CI: 86–100%) were observed in the 25 evaluable patients who received the MTD. Median OS was 19.6 months in 29 assessable patients (95% CI: 15.5–27.2) (Figure 1A) and median TTP was 8.5 months in 27 assessable patients (95% CI: 7.6–11.1) (Figure 1B). One-year survival was 72.4% (95% CI: 52.3–85.1%) and 2-year survival was 37.2% (95% CI: 13.2–61.8%).

The ORR in the 38 evaluable patients in the phase I/II study was 63% (95% CI: 46–78%), with a disease-control rate of 89% (95% CI: 75–97%). After a median follow-up of 14.9 months, median OS was 16.8 months (95% CI: 9.6–25.3) in 45 evaluable patients (Figure 1C) and median TTP was 8.7 months (95% CI: 7.9–10.4) in 40 evaluable patients (Figure 1D). One-year survival was 62.2% (95% CI: 46.4–74.6%) and 2-year survival was 38.7% (95% CI: 21.6–55.5%).

Liver resections were performed on three patients. One patient with a partial response after four cycles underwent an R0 resection, one patient who had stable disease after three cycles had an R1 resection and one patient who had 5 cycles and achieved a partial response had an R1 resection. Another patient, who underwent a total of 11 treatment cycles and achieved a partial response on a re-challenge with SCOUT is being assessed for liver resection.

### Tolerability

Tolerability data for the phase I cohort are detailed in our earlier publication. In the MTD group, 138 UFT courses were prescribed to the 29 patients, 16 of which were reduced by 1 capsule daily and 8 of which were reduced by 2 capsules daily; 21 patients (72%) received UFT without a dose reduction, 13 of whom (45%) received all 6 UFT cycles at full dose. A total of 139 doses of irinotecan therapy were administered, 17 doses were reduced by 20% and 3 by 50%; 132 doses of oxaliplatin were administered, 9 were reduced by 20% and 5 by 50%. The median dose intensity was 89% for both irinotecan and oxaliplatin after two cycles; 92% and 91% for irinotecan and oxaliplatin, respectively, after four cycles; and 95% and 92% for irinotecan and oxaliplatin, respectively, after six cycles.

SCOUT was well tolerated at the MTD, as shown in Table 4. One patient developed grade 3 neutropaenia and another had grade 3 leucopaenia. One patient with a history of hypertension, atrial fibrillation and type II diabetes had a grade 4 cardiac event and died following a myocardial infarction before cycle four. Alopecia and neurotoxicity were minimal: three patients (10%) had grade 2 alopecia and three (10%) had grade 2/3 neurotoxicity. Hand–foot syndrome grade >1 was not observed.

### Second-line therapy

After failure of first-line therapy, 24 out of the 29 patients (83%) treated at the MTD were considered for second-line treatment; 3 patients had not progressed at the time of the analysis and a further 2 patients were excluded as they were not assessable for progression, as described above. Six patients (25%) were deemed unfit to receive second-line therapy. Eighteen patients received second-line therapy (Table 5). Nine patients (38%) resumed SCOUT, eight of whom were assessable for response: one patient had a complete response (13%); three had partial responses (38%); one had stable disease (13%) and three had progressive disease (38%). Therefore, the ORR to re-treatment with SCOUT in the MTD group was 50% and the disease control rate was 63%. Twelve patients received other second-line regimens, including capecitabine-, irinotecan- and oxaliplatin-based regimens, as detailed in Table 5.

If patients treated at all doses are considered, 36 out of the 45 patients (80%) were considered for second-line therapy, 12 (33%) of whom were deemed unfit for further chemotherapy. Twelve (50%) of the 24 patients who received second-line chemotherapy resumed SCOUT. Two of the additional three patients from the phase I study who resumed SCOUT responded to the re-challenge with stable disease, and one had progressive disease. Therefore, the ORR to re-treatment with SCOUT chemotherapy in all patients was 36% and the disease control rate was 64%.

Nine out of the 12 patients (75%) who received second-line SCOUT went on to receive a third-line chemotherapy regimen, as shown in Table 5.

Out of the remaining 12 patients who received second-line therapies other than SCOUT, 3 (25%) went on to receive a third-line agent (mitomycin C/capecitabine: *n*=2; XELOX: *n*=1).

## Discussion

Recent studies have demonstrated that triple-drug regimens can improve survival and response in patients with mCRC. Many of these regimens, however, can be associated with excessive toxicities. Alternating therapy, in which only two out of the three drugs are administered at one time, provides a means of delivering three effective drugs while minimising toxicity. The results from the present study confirm that alternating irinotecan and oxaliplatin during treatment with UFT with LV allows patients to receive an effective triple-drug regimen without the excessive haematological toxicities often observed with such treatments.

The combination of UFT with LV plus irinotecan and oxaliplatin was highly effective, with excellent ORRs that were consistent with the phase I study results and better than those reported for UFT with LV plus irinotecan (Mendez *et al*, 2005; Delord *et al*, 2007; Bajetta *et al*, 2007a) or UFT with LV plus oxaliplatin (Feliu *et al*, 2004; Rosati *et al*, 2005; Bennouna *et al*, 2006; Bajetta *et al*, 2007a), providing further support for the up-front treatment approach. Particularly notable was the fact that all patients treated at the MTD benefited from therapy, with partial responses in 68% patients and stable disease in 32%. The OS of 19.6 months seen in patients treated at the MTD was comparable with results obtained for other triplet regimens (Table 1).

Alternating oxaliplatin and irinotecan every 2 weeks allowed relatively high doses of both agents to be used without significant toxicities. Alopecia and neurotoxicity, which are commonly observed in patients treated with irinotecan and oxaliplatin, were minimal in our patients. Grade 2 alopecia was observed in three patients (7%) and only one patient had grade 3 neuropathy. Another feature of this study was the very low incidence of grade 3/4 haematological toxicities. This is likely to be a result of the alternating schedule, in which a 4-week gap between each dose of irinotecan and each dose of oxaliplatin allowed patients to recover from oxaliplatin- and irinotecan-induced toxicities before each drug was administered again. Indeed, at the MTD, only one patient had grade 3 neuropathy. Grade 3/4 toxicities were mainly gastrointestinal, with grade 3 diarrhoea in 10% patients. The single grade 4 adverse event observed at the MTD was an unrelated cardiac event in a patient with a history of hypertension, atrial fibrillation and type II diabetes. Hand–foot syndrome, a disturbing and disabling condition that can impact the quality of life of affected patients (Scheithauer and Blum, 2004), is often seen in patients treated with 5-FU and capecitabine, but was not observed at grades >1 in the present study. This study was small, however, and larger studies would be required to allow definitive conclusions to be drawn regarding the propensity of UFT to cause this syndrome.

UFT is a convenient alternative to infusional 5-FU, as it does not require the use of Hickman lines or pumps and therefore the complications associated with these modes of administration can be avoided (Puig-la Calle *et al*, 1996). Studies have shown that patients prefer UFT to i.v. 5-FU regimens, with most patients citing the convenience of oral treatment as a reason for this preference (Borner *et al*, 2002; Rocha Lima and del Giglio, 2005). However, patient preference is largely driven by tolerability, as shown by a recent study comparing capecitabine with the Nordic 5-FU/LV regimen (Pfeiffer *et al*, 2006). In that study, i.v. 5-FU was preferred to oral capecitabine, primarily because capecitabine-treated patients had a higher incidence of adverse events, including diarrhoea and hand–foot syndrome, whereas the Nordic 5-FU regimen was well tolerated.

One concern regarding the use of intensive up-front triple therapy is that the choice of second-line treatment may be limited when patients progress. This is not the case with SCOUT, as the tolerability of the alternating regimen meant that most patients (67% of those in the phase I/II study group) were able to receive further treatment, including repeated courses of SCOUT. Re-treatment with SCOUT, which occurred in 33% patients eligible for second-line therapy, resulted in further complete and partial responses. This is in agreement with a previous report that first-line FOLFOXIRI (5-FU, oxaliplatin and irinotecan) did not compromise the feasibility of second-line treatments (Masi *et al*, 2006). In that study, 76% patients received second-line therapy and further responses were observed in 33% patients, similar to our findings. By virtue of their disease being chemosensitive, most patients (75%) who were suitable for second-line SCOUT went on to receive a third-line regimen, in contrast to only 25% of those who resumed alternate second-line chemotherapy. Again, this lends support to the good tolerability of SCOUT as well as to the concept that further alternate lines of chemotherapy are still possible after SCOUT.

Other triple-drug regimens have been shown to result in excellent ORRs and survival. Objective response rates of 43–72% and OS of 21.5–28.4 months have been reported for FOLFOXIRI (Masi *et al*, 2004; Souglakos *et al*, 2006; Falcone *et al*, 2007). Variations in response and survival outcomes are likely to be a result of differences in the regimens used, for example, the doses of irinotecan, oxaliplatin and 5-FU/LV were higher in the study by Falcone and colleagues. In addition, the characteristics of patients entered into the study also differed: patients in the study by Souglakos and colleagues were not required to be younger than 75 years and the proportion of patients with Eastern Cooperative Oncology Group performance status 2 was considerably higher (50% compared with 35% in the Hellenic Group study). The triple-drug combination of capecitabine, oxaliplatin and irinotecan (XELOXIRI) has also been investigated (Masi *et al*, 2007; Bajetta *et al*, 2007b). Efficacy outcomes were good, with an ORR of 63% and median OS of 23.5 months in the study by Bajetta and colleages, and an ORR of 70% and median progression-free survival of 9.2 months in the study by Masi and colleagues. FOLFOXIRI and XELOXIRI, however, caused considerable haematological toxicity, neurotoxicity and diarrhoea. Grade 3/4 neutropaenia appears to be a particularly common problem with concurrent triple-drug therapy, although this was rare with the SCOUT regimen (grade 3 neutropaenia occurred in only 3% patients).

Others have used 5-FU- and capecitabine-based alternating regimens with good results. Objective response rates of 46–54% and OS of 18–18.7 months have been observed in alternating 5-FU-based regimens, along with little neurotoxicity and myelotoxicity (Aparicio *et al*, 2005; Ferrari *et al*, 2005; Hebbar *et al*, 2006). Cassinello *et al* (2006) reported a somewhat lower ORR of 37% and OS of 16.4 months for a regimen comprising oxaliplatin on day 1 with capecitabine on days 1–14 of a 21-day cycle, followed by irinotecan on day 1 plus capecitabine on days 1–14 of a second 21-day cycle. Nonetheless, this regimen was well tolerated, providing further support for the improved tolerability of alternating regimens.

Considerable debate surrounds the optimal approach to treating patients with mCRC. Results from the CAIRO and FOCUS studies suggest that first-line monotherapy followed by second-line combination therapy is as effective as combination chemotherapy in the first line followed by monotherapy (Koopman *et al*, 2007; Seymour *et al*, 2007). In contrast, OPTIMOX1 indicated that intensive first-line therapy with prolonged maintenance treatment and planned reintroduction of intensive therapy is a valid approach to treatment (Tournigand *et al*, 2006). The OPTIMOX2 study has demonstrated, however, that a break in therapy between intensive treatments cannot be recommended (Maindrault-Goebel *et al*, 2007). The SCOUT approach avoids the need for discontinuing oxaliplatin, although reducing treatment intensity between full-dose courses may be attractive to some patients.

Several limitations of this study need to be considered. This was a single-centre, non-randomised study, making direct comparisons with other studies difficult, and the patient numbers were small. In addition, this study did not include a targeted agent, such as cetuximab or bevacizumab, and therefore the question whether targeted agents might improve response and survival rates remains unanswered. However, it can be argued that the first priority in combination chemotherapy scheduling should be optimisation of the three well-known, conventional, active chemotherapy agents in mCRC. As stated by Saltz at ASCO in 2002, ‘we need all three drugs. We have conflicting data about how to best use them, but, clearly, we need to have them all available to our patients’. Evaluation of the role of the targeted agents combined with a well-tolerated and effective alternating regimen, like SCOUT, would be the next key step to improve outcomes further. Such a study is underway, in which the SCOUT regimen will be combined with cetuximab (E-SCOUT).

In conclusion, UFT with LV plus alternating irinotecan and oxaliplatin is an effective, well-tolerated treatment approach for patients with mCRC. SCOUT results in high ORRs (63%, 95% CI: 49–80), respectable survival (median TTP and OS were 8.7 months (95% CI: 7.9–10.4) and 16.8 months (95% CI: 9.6–25.3), respectively), and the feasibility of second- and third-line treatments. SCOUT is convenient for both patients and hospital staff, as it avoids the need for tunnelled central lines and their associated complications, translating into saving out-patient time, in-patient admissions and clinical costs.

## Figures and Tables

**Table 1:** Patients receiving second-line therapy in randomised clinical studies (adapted from Grothey *et al*, 2004)

**First-line regimen**	**Patients with second-line therapy (%)**	**Patients administered three active drugs (%)**	**Median overall survival (months)**	**Reference**
Irinotecan+bolus 5-FU/LV	52	5	14.8	Saltz *et al* (2000)
Irinotecan+bolus 5-FU/LV	67	24	15.0	Goldberg *et al* (2004)
Irinotecan+CIV 5-FU/LV	39	16	17.4	Douillard *et al* (2000)
Irinotecan+AIO	56	52	20.1	Köhne *et al* (2005)
Irinotecan+CIV 5-FU/LV	81	74	21.5	Tournigand *et al* (2004)
FOLFOX4	58	30	16.2	de Gramont *et al* (2000)
FOLFOX4	75	60	19.5	Goldberg *et al* (2004)
FOLFOX6	62	74	20.6	Tournigand *et al* (2004)
FOLFOX7	73	61	21.2	Tournigand *et al* (2006)

AIO=Arbeitsgemeinschaft für Internistische Onkologie; CIV=continuous intravenous infusion; FOLFOX=5-FU/LV+oxaliplatin; 5-FU=5-fluorouracil; LV=Leucovorin®.

**Table 2:** Patient characteristics at baseline (patients treated at the MTD)

**Characteristic**	**Value**
No. of patients	29
Median age (range) (years)	57 (40–73)
	
*Sex,* n *(%)*	
Male	25 (86)
Female	4 (14)
	
*Primary tumour site,* n *(%)*	
Colon	15 (52)
Rectum	9 (31)
Rectosigmoid	5 (17)
	
*WHO performance status,* n *(%)*	
0	15 (52)
1	11 (38)
2	1 (3)
Unknown	2 (7)
	
*Prior surgical resection,* n *(%)*	
Radical	9 (31)
Palliative	10 (34)
Defunctioned only	2 (7)
None	8 (28)
	
*No. of measurable lesions (%)*	
1	1 (3)
2	7 (24)
⩾3	21 (72)

MTD=maximum tolerated dose; WHO=World Health Organization.

**Table 3:** Anti-tumour efficacy of SCOUT

**Outcome, *n* (%)**	**Patients at all doses (*n*=45)**	**MTD patients (*n*=29)**
Not assessable^a^	7	4
Evaluable patients	38	25
Complete response, *n* (%)^b^	0	0
Partial response, *n* (%)^b^	24 (63)	17 (68)
Stable disease, *n* (%)^b^	10 (26)	8 (32)
Disease control rate (95% CI)^c^	89 (75–97)	100 (86–100)
Progressive disease, *n* (%)^b^	4 (11)	0
Objective response rate (95% CI)	63 (46–78)	68 (46–85)

CI=confidence interval; MTD=maximum tolerated dose.

a Patients did not receive two treatment cycles and were thus not assessable for response.

b Based on the number of evaluable patients.

c Complete response+partial response+stable disease rates.

**Table 4:** Grade 3/4 adverse events per patient treated at the MTD (all cycles)

	**NCI-CTC grade, *n* (%) (*N*=29)**	
**Event**	**3**	**4**
*Haematological*		
Anaemia	0	0
Leucopaenia	1 (3)	0
Neutropaenia	1 (3)	0
Thrombocytopaenia	0	0
		
*Non-haematological*		
Alopecia	NA	NA
Anorexia	0	0
Lethargy	1 (3)	0
Nausea	0	0
Vomiting	0	0
Diarrhoea	3 (10)	0
Constipation	0	0
AST elevation	1 (3)	0
Neuropathy	1 (3)	0
Hand–foot syndrome	0	0
Infection	0	0
Abdominal pain	2 (7)	0
Cardiac	0	1 (3)

AST=aspartate aminotransferase; MTD=maximum tolerated dose; NA=not applicable; NCI-CTC=National Cancer Institute Common Toxicity Criteria.

**Table 5:** Second- and third-line therapy following SCOUT

**Regimen**	**No. of patients, *n* (%), at all doses (*N*=45)**	**No. of patients, *n* (%), at MTD (*N*=29)**
*Second-line therapy*		
Eligible for second-line therapy^a^	36	24
Resumed SCOUT	12 (33)	9 (38)
Mitomycin C/capecitabine	3 (8)	2 (8)
Irinotecan/cetuximab	3 (8)	3 (13)
Oxaliplatin/modified de Gramont	1 (3)	1 (4)
Oxaliplatin/capecitabine	1 (3)	0
Capecitabine	1 (3)	0
Phase I trial	3 (8)	3 (13)
Unfit for further chemotherapy	12 (33)	6 (25)
		
	**No. of patients at all doses (*N*=45)**	
*Third-line therapy*		
Second-line SCOUT patients eligible for third-line therapy^b^	12	
Resumed SCOUT	2	
Mitomycin C/capecitabine	1	
Irinotecan/cetuximab	1	
Capecitabine	1	
Phase I trial	4	
Unfit for further chemotherapy	2	
Not progressed on second-line therapy	1	

MTD=maximum tolerated dose.

a Excludes patients who were not assessable for progression and those who had not progressed at the time of the analysis.

b Patients who received SCOUT as second-line therapy and were considered eligible for further treatment; excludes patients who had second-line treatments other than SCOUT.

## References

[bib1] Aparicio J, Fernandez-Martos C, Vincent JM, Maestu I, Llorca C, Busquier I, Campos JM, Perez-Enguix D, Balcells M (2005) FOLFOX alternated with FOLFIRI as first-line chemotherapy for metastatic colorectal cancer. Clin Colorectal Cancer 5: 263–267 16356303 10.3816/ccc.2005.n.037

[bib2] Bajetta E, Celio L, Ferrario E, Di Bartolomeo M, Denaro A, Dotti K, Mancin M, Bajetta R, Colombo A, Pusceddu S (2007b) Capecitabine plus oxaliplatin and irinotecan regimen every other week: a phase I/II study in first-line treatment of metastatic colorectal cancer. Ann Oncol 18: 1810–1816 17823385 10.1093/annonc/mdm347

[bib3] Bajetta E, Di Bartolomeo M, Buzzoni R, Mariani L, Zilembo N, Ferrario E, Vullo SL, Aitini E, Isa L, Barone C, Jacobelli S, Recaldin E, Pinotti G, Iop A (2007a) Uracil/ftorafur/leucovorin combined with irinotecan (TEGAFIRI) or oxaliplatin (TEGAFOX) as first-line treatment for metastatic colorectal cancer patients: results of randomised phase II study. Br J Cancer 96: 439–444 17245343 10.1038/sj.bjc.6603493 PMC2360030

[bib4] Bennouna J, Perrier H, Paillot B, Priou F, Jacob JH, Hebbar M, Bordenave S, Seitz JF, Cvitkovic F, Dorval E, Malek K, Tonelli D, Douillard JY (2006) A phase II study of oral uracil/ftorafur (UFT) plus leucovorin combined with oxaliplatin (TEGAFOX) as first-line treatment in patients with metastatic colorectal cancer. Br J Cancer 94: 69–73 16404362 10.1038/sj.bjc.6602913 PMC2361076

[bib5] Borner MM, Schoffski P, de Wit R, Caponigro F, Comella G, Sulkes A, Greim G, Peters GJ, van der Born K, Wanders J, de Boer RF, Martin C, Fumoleau P (2002) Patient preference and pharmacokinetics of oral modulated UFT versus intravenous fluorouracil and leucovorin: a randomised crossover trial in advanced colorectal cancer. Eur J Cancer 38: 349–358 11818199 10.1016/s0959-8049(01)00371-9

[bib6] Carmichael J, Popiela T, Radstone D, Falk S, Borner M, Oza A, Skovsgaard T, Munier S, Martin C (2002) Randomized comparative study of tegafur/uracil and oral leucovorin versus parenteral fluorouracil and leucovorin in patients with previously untreated metastatic colorectal cancer. J Clin Oncol 20: 3617–3627 12202662 10.1200/JCO.2002.10.129

[bib7] Cassinello J, Alvarez JV, Lopez MJ, Pujol E, Colmenarejo A, Segovia F, Marcos F, Filipovich E, Arcediano A, Castro IG (2006) Multicenter phase II study of fixed sequences of capecitabine combined with oxaliplatin or irinotecan in patients with previously untreated metastatic colorectal cancer. Clin Colorectal Cancer 5: 429–435 16635282 10.3816/ccc.2006.n.014

[bib8] de Gramont A, Figer A, Seymour M, Homerin M, Hmissi A, Cassidy J, Boni C, Cortes-Funes H, Cervantes A, Freyer G, Papamichael D, Le Bail N, Louvet C, Hendler D, de Braud F, Wilson C, Morvan F, Bonetti A (2000) Leucovorin and fluorouracil with or without oxaliplatin as first-line treatment in advanced colorectal cancer. J Clin Oncol 18: 2938–2947 10944126 10.1200/JCO.2000.18.16.2938

[bib9] Delord JP, Bennouna J, Artru P, Perrier H, Husseini F, Desseigne F, Francois E, Faroux R, Smith D, Piedbois P, Naman H, Douillard JY, Bugat R (2007) Phase II study of UFT with leucovorin and irinotecan (TEGAFIRI): first-line therapy for metastatic colorectal cancer. Br J Cancer 97: 297–301 17637682 10.1038/sj.bjc.6603889 PMC2360336

[bib10] Douillard JY, Cunningham D, Roth AD, Navarro M, James RD, Karasek P, Jandik P, Iveson T, Carmichael J, Alakl M, Gruia G, Awad L, Rougier P (2000) Irinotecan combined with fluorouracil compared with fluorouracil alone as first-line treatment for metastatic colorectal cancer: a multicentre randomised trial. Lancet 355: 1041–1047 10744089 10.1016/s0140-6736(00)02034-1

[bib11] Douillard JY, Hoff PM, Skillings JR, Eisenberg P, Davidson N, Harper P, Vincent MD, Lembersky BC, Thompson S, Maniero A, Benner SE (2002) Multicenter phase III study of uracil/tegafur and oral leucovorin versus fluorouracil and leucovorin in patients with previously untreated metastatic colorectal cancer. J Clin Oncol 20: 3605–3616 12202661 10.1200/JCO.2002.04.123

[bib12] Falcone A, Ricci S, Brunetti I, Pfanner E, Allegrini G, Barbara C, Crino L, Benedetti G, Evangelista W, Fanchini L, Cortesi E, Picone V, Vitello S, Chiara S, Granetto C, Porcile G, Fioretto L, Orlandini C, Andreuccetti M, Masi G (2007) Phase III trial of infusional fluorouracil, leucovorin, oxaliplatin, and irinotecan (FOLFOXIRI) compared with infusional fluorouracil, leucovorin, and irinotecan (FOLFIRI) as first-line treatment for metastatic colorectal cancer: the Gruppo Oncologico Nord Ovest. J Clin Oncol 25: 1670–1676 17470860 10.1200/JCO.2006.09.0928

[bib13] Feliu J, Vicent JM, Garcia-Giron C, Constela M, Fonseca E, Aparicio J, Lomas M, Anton-Aparicio L, Dorta FJ, Gonzalez Baron M (2004) Phase II study of UFT and oxaliplatin in first-line treatment of advanced colorectal cancer. Br J Cancer 91: 1758–1762 15505621 10.1038/sj.bjc.6602217 PMC2410059

[bib14] Ferrari V, Valcamonico F, Amoroso V, Simoncini E, Vassalli L, Marpicati P, Rangoni G, Grisanti S, Pasinetti N, Marini G (2005) An alternating regimen of irinotecan/5-fluorouracil/folinic acid and oxaliplatin/5-fluorouracil/folinic acid in metastatic colorectal cancer: a phase II trial. Oncology 69: 283–289 16282707 10.1159/000089677

[bib15] Goldberg RM, Sargent DJ, Morton RF, Fuchs CS, Ramanathan RK, Williamson SK, Findlay BP, Pitot HC, Alberts SR (2004) A randomized controlled trial of fluorouracil plus leucovorin, irinotecan, and oxaliplatin combinations in patients with previously untreated metastatic colorectal cancer. J Clin Oncol 22: 23–30 14665611 10.1200/JCO.2004.09.046

[bib16] Grothey A, Sargent D (2005) Overall survival of patients with advanced colorectal cancer correlates with availability of fluorouracil, irinotecan, and oxaliplatin regardless of whether doublet or single-agent therapy is used first line. J Clin Oncol 23: 9441–9442 16361649 10.1200/JCO.2005.04.4792

[bib17] Grothey A, Sargent D, Goldberg RM, Schmoll HJ (2004) Survival of patients with advanced colorectal cancer improves with the availability of fluorouracil-leucovorin, irinotecan, and oxaliplatin in the course of treatment. J Clin Oncol 22: 1209–1214 15051767 10.1200/JCO.2004.11.037

[bib18] Hebbar M, Tournigand C, Lledo G, Mabro M, Andre T, Louvet C, Aparicio T, Flesch M, Varette C, de Gramont A (2006) Phase II trial alternating FOLFOX-6 and FOLFIRI regimens in second-line therapy of patients with metastatic colorectal cancer (FIREFOX study). Cancer Invest 24: 154–159 16537184 10.1080/07357900500524397

[bib19] Köhne CH, van Cutsem E, Wils J, Bokemeyer C, El-Serafi M, Lutz MP, Lorenz M, Reichardt P, Ruckle-Lanz H, Frickhofen N, Fuchs R, Mergenthaler HG, Langenbuch T, Vanhoefer U, Rougier P, Voigtmann R, Muller L, Genicot B, Anak O, Nordlinger B (2005) Phase III study of weekly high-dose infusional fluorouracil plus folinic acid with or without irinotecan in patients with metastatic colorectal cancer: European Organisation for Research and Treatment of Cancer Gastrointestinal Group Study 40986. J Clin Oncol 23: 4856–4865 15939923 10.1200/JCO.2005.05.546

[bib20] Koopman M, Antonini NF, Douma J, Wals J, Honkoop AH, Erdkamp FL, de Jong RS, Rodenburg CJ, Vreugdenhil G, Loosveld OJ, van Bochove A, Sinnige HA, Creemers GJ, Tesselaar ME, Slee PH, Werter MJ, Mol L, Dalesio O, Punt CJ (2007) Sequential versus combination chemotherapy with capecitabine, irinotecan, and oxaliplatin in advanced colorectal cancer (CAIRO): a phase III randomised controlled trial. Lancet 370: 135–142 17630036 10.1016/S0140-6736(07)61086-1

[bib21] Maindrault-Goebel F, Lledo G, Chibaydel B, Mineur L, Andre T, Mabro M, Artru P, Louvet C, de Gramont A (2007) Final results of OPTIMOX2, a large randomized phase II study of maintenance therapy or chemotherapy-free intervals (CFI) after FOLFOX in patients with metastatic colorectal cancer (MRC): a GERCOR study. J Clin Oncol 25[18S (Suppl)]: 4013 (abstr)

[bib22] Masi G, Allegrini G, Cupini S, Marcucci L, Cerri E, Brunetti I, Fontana E, Ricci S, Andreuccetti M, Falcone A (2004) First-line treatment of metastatic colorectal cancer with irinotecan, oxaliplatin and 5-fluorouracil/leucovorin (FOLFOXIRI): results of a phase II study with a simplified biweekly schedule. Ann Oncol 15: 1766–1772 15550581 10.1093/annonc/mdh470

[bib23] Masi G, Barletta M, Baldi GG, Antonuzzo A, Sonaglio C, Pfanner E, Petrini I, Falcone A (2007) The combination of capecitabine (C), irinotecan (I) and oxaliplatin (O) (XELOXIRI) as first line treatment of metastatic colorectal cancer (MCRC): preliminary results of a pilot study by the Gruppo Oncologico Nord-Ovest (G.O.N.O.). J Clin Oncol 25[18S (Suppl)]: 4096 (abstr)17827459

[bib24] Masi G, Marcucci L, Loupakis F, Cerri E, Barbara C, Bursi S, Allegrini G, Brunetti IM, Murr R, Ricci S, Cupini S, Andreuccetti M, Falcone A (2006) First-line 5-fluorouracil/folinic acid, oxaliplatin and irinotecan (FOLFOXIRI) does not impair the feasibility and the activity of second line treatments in metastatic colorectal cancer. Ann Oncol 17: 1249–1254 16766580 10.1093/annonc/mdl119

[bib25] Mendez M, Alfonso PG, Pujol E, Gonzalez E, Castanon C, Cerezuela P, Lopez-Mateos Y, Cruz JJ (2005) Weekly irinotecan plus UFT and leucovorin as first-line chemotherapy of patients with advanced colorectal cancer. Invest New Drugs 23: 243–251 15868381 10.1007/s10637-005-6733-0

[bib26] Meta-Analysis Group In Cancer (1998) Toxicity of fluorouracil in patients with advanced colorectal cancer: effect of administration schedule and prognostic factors. Meta-Analysis Group In Cancer. J Clin Oncol 16: 3537–3541 9817272 10.1200/JCO.1998.16.11.3537

[bib27] Pfeiffer P, Mortensen JP, Bjerregaard B, Eckhoff L, Schonnemann K, Sandberg E, Aabo K, Jakobsen A (2006) Patient preference for oral or intravenous chemotherapy: a randomised cross-over trial comparing capecitabine and Nordic fluorouracil/leucovorin in patients with colorectal cancer. Eur J Cancer 42: 2738–2743 17011184 10.1016/j.ejca.2006.06.027

[bib28] Puig-la Calle Jr J, Lopez SS, Piedrafita SE, Allende HL, Artigas RV, Puig la CJ (1996) Totally implanted device for long-term intravenous chemotherapy: experience in 123 adult patients with solid neoplasms. J Surg Oncol 62: 273–278 8691841 10.1002/(SICI)1096-9098(199608)62:4<273::AID-JSO9>3.0.CO;2-3

[bib29] Rocha Lima AP, del Giglio A (2005) Randomized crossover trial of intravenous 5-FU versus oral UFT both modulated by leucovorin: a one-centre experience. Eur J Cancer Care (Engl) 14: 151–154 15842464 10.1111/j.1365-2354.2005.00531.x

[bib30] Rosati G, Cordio S, Tucci A, Blanco G, Bordonaro R, Reggiardo G, Manzione L (2005) Phase II trial of oxaliplatin and tegafur/uracil and oral folinic acid for advanced or metastatic colorectal cancer in elderly patients. Oncology 69: 122–129 16118508 10.1159/000087814

[bib31] Saltz LB, Cox JV, Blanke C, Rosen LS, Fehrenbacher L, Moore MJ, Maroun JA, Ackland SP, Locker PK, Pirotta N, Elfring GL, Miller LL (2000) Irinotecan plus fluorouracil and leucovorin for metastatic colorectal cancer. Irinotecan Study Group. N Engl J Med 343: 905–914 11006366 10.1056/NEJM200009283431302

[bib32] Scheithauer W, Blum J (2004) Coming to grips with hand–foot syndrome. Insights from clinical trials evaluating capecitabine. Oncology (Williston Park) 18: 1161–1168, 1173 15471200

[bib33] Seymour MT, Maughan TS, Ledermann JA, Topham C, James R, Gwyther SJ, Smith DB, Shepherd S, Maraveyas A, Ferry DR, Meade AM, Thompson L, Griffiths GO, Parmar MK, Stephens RJ (2007) Different strategies of sequential and combination chemotherapy for patients with poor prognosis advanced colorectal cancer (MRC FOCUS): a randomised controlled trial. Lancet 370: 143–152 17630037 10.1016/S0140-6736(07)61087-3

[bib34] Sheikh HY, Valle JW, Palmer K, Sjursen A, Craven O, Wilson G, Swindell R, Saunders MP (2007) Concurrent irinotecan, oxaliplatin and UFT in first-line treatment of metastatic colorectal cancer: a phase I study. Br J Cancer 96: 38–43 17213824 10.1038/sj.bjc.6603521 PMC2360216

[bib35] Souglakos J, Androulakis N, Syrigos K, Polyzos A, Ziras N, Athanasiadis A, Kakolyris S, Tsousis S, Kouroussis C, Vamvakas L, Kalykaki A, Samonis G, Mavroudis D, Georgoulias V (2006) FOLFOXIRI (folinic acid, 5-fluorouracil, oxaliplatin and irinotecan) vs FOLFIRI (folinic acid, 5-fluorouracil and irinotecan) as first-line treatment in metastatic colorectal cancer (MCC): a multicentre randomised phase III trial from the Hellenic Oncology Research Group (HORG). Br J Cancer 94: 798–805 16508637 10.1038/sj.bjc.6603011 PMC2361370

[bib36] Therasse P, Arbuck SG, Eisenhauer EA, Wanders J, Kaplan RS, Rubinstein L, Verweij J, Van GM, van Oosterom AT, Christian MC, Gwyther SG (2000) New guidelines to evaluate the response to treatment in solid tumors. European Organization for Research and Treatment of Cancer, National Cancer Institute of the United States, National Cancer Institute of Canada. J Natl Cancer Inst 92: 205–216 10655437 10.1093/jnci/92.3.205

[bib37] Tournigand C, Andre T, Achille E, Lledo G, Flesh M, Mery-Mignard D, Quinaux E, Couteau C, Buyse M, Ganem G, Landi B, Colin P, Louvet C, de Gramont A (2004) FOLFIRI followed by FOLFOX6 or the reverse sequence in advanced colorectal cancer: a randomized GERCOR study. J Clin Oncol 22: 229–237 14657227 10.1200/JCO.2004.05.113

[bib38] Tournigand C, Cervantes A, Figer A, Lledo G, Flesch M, Buyse M, Mineur L, Carola E, Etienne PL, Rivera F, Chirivella I, Perez-Staub N, Louvet C, Andre T, Tabah-Fisch I, de Gramont A (2006) OPTIMOX1: a randomized study of FOLFOX4 or FOLFOX7 with oxaliplatin in a stop-and-go fashion in advanced colorectal cancer – a GERCOR study. J Clin Oncol 24: 394–400 16421419 10.1200/JCO.2005.03.0106

[bib39] Verso M, Agnelli G (2003) Venous thromboembolism associated with long-term use of central venous catheters in cancer patients. J Clin Oncol 21: 3665–3675 14512399 10.1200/JCO.2003.08.008

